# Real-time detection of colon polyps during colonoscopy using deep learning: systematic validation with four independent datasets

**DOI:** 10.1038/s41598-020-65387-1

**Published:** 2020-05-20

**Authors:** Ji Young Lee, Jinhoon Jeong, Eun Mi Song, Chunae Ha, Hyo Jeong Lee, Ja Eun Koo, Dong-Hoon Yang, Namkug Kim, Jeong-Sik Byeon

**Affiliations:** 1 Health Screening and Promotion Center, Asan Medical Center, Seoul, Republic of Korea; 2 Department of Biomedical Engineering, Asan Medical Institute of Convergence Science and Technology, Asan Medical Center, University of Ulsan College of Medicine, Seoul, Republic of Korea; 3 Department of Gastroenterology, Asan Medical Center, University of Ulsan College of Medicine, Seoul, Republic of Korea; 4 Department of Convergence Medicine, University of Ulsan College of Medicine, Asan Medical Center, Seoul, Republic of Korea

**Keywords:** Computational biology and bioinformatics, Gastroenterology

## Abstract

We developed and validated a deep-learning algorithm for polyp detection. We used a YOLOv2 to develop the algorithm for automatic polyp detection on 8,075 images (503 polyps). We validated the algorithm using three datasets: A: 1,338 images with 1,349 polyps; B: an open, public CVC-clinic database with 612 polyp images; and C: 7 colonoscopy videos with 26 polyps. To reduce the number of false positives in the video analysis, median filtering was applied. We tested the algorithm performance using 15 unaltered colonoscopy videos (dataset D). For datasets A and B, the per-image polyp detection sensitivity was 96.7% and 90.2%, respectively. For video study (dataset C), the per-image polyp detection sensitivity was 87.7%. False positive rates were 12.5% without a median filter and 6.3% with a median filter with a window size of 13. For dataset D, the sensitivity and false positive rate were 89.3% and 8.3%, respectively. The algorithm detected all 38 polyps that the endoscopists detected and 7 additional polyps. The operation speed was 67.16 frames per second. The automatic polyp detection algorithm exhibited good performance, as evidenced by the high detection sensitivity and rapid processing. Our algorithm may help endoscopists improve polyp detection.

## Introduction

Colonoscopy is an important colorectal cancer (CRC) screening test worldwide. Colonoscopy has several advantages, such as the removal of lesions and visualization in a single test. Recent studies indicated that having a colonoscopy was associated with a 60% reduction in CRC mortality^1^ and a 70% reduction in the incidence of late-stage CRCs^2^.

Colonoscopy quality assurance is of paramount importance for effective prevention of CRC and reduction of mortality due to CRC. Accurate detection of adenomas is the most critical issue during a colonoscopy. The adenoma detection rate is an essential quality indicator during colonoscopy. Evidence suggests that a 1.0% increase in the adenoma detection rate leads to a 3.0% decrease in the risk of interval CRC^3^. The adenoma detection rate varies from 17% to 47% because the characteristics of colonoscopy are highly operator-dependent^4^. Therefore, it is important to increase the adenoma detection rate for adequate CRC screening via colonoscopy.

Although many efforts have been directed toward improving the detection of adenoma, such as improving the bowel preparation, spending enough time to inspect the colonic mucosa, and developing several novel technologies, such as wide-angle cameras and cap-assisted techniques to flatten colonic folds^5^, the problem of missing polyps remains. A previous study indicated that endoscopists with wider visual gaze patterns achieved a higher polyp detection rate than those with centralized visual gaze patterns^6^. Several studies have indicated that the participation of an experienced nurse during the colonoscopy examination as a “second observer” increased the adenoma detection rates by up to 30–50%^7,8^ and increased the detection performance of inexperienced endoscopists^7^. A real-time automatic polyp detection system has the potential to compensate for limitations of the visual field of endoscopists, similar to a second observer; the system would indicate suspected areas on the monitor and draw the endoscopists’ visual attention to the region of interest. Automatic polyp detection systems using deep-learning methods have been proposed for detecting colorectal polyps in real-time colonoscopy videos ^9–11^. Despite the optimistic results of previous studies, further investigations are necessary to show the generalizability of deep-learning algorithms. Therefore, we developed a deep-learning algorithm to confirm the feasibility of an artificial intelligence system for automatic polyp detection during colonoscopy. We tested the performance of the algorithm using unaltered colonoscopy videos after systematic validation using two datasets of still images and one independent video dataset.

## Results

### Validation of algorithm using three different datasets

**Figure 1:**
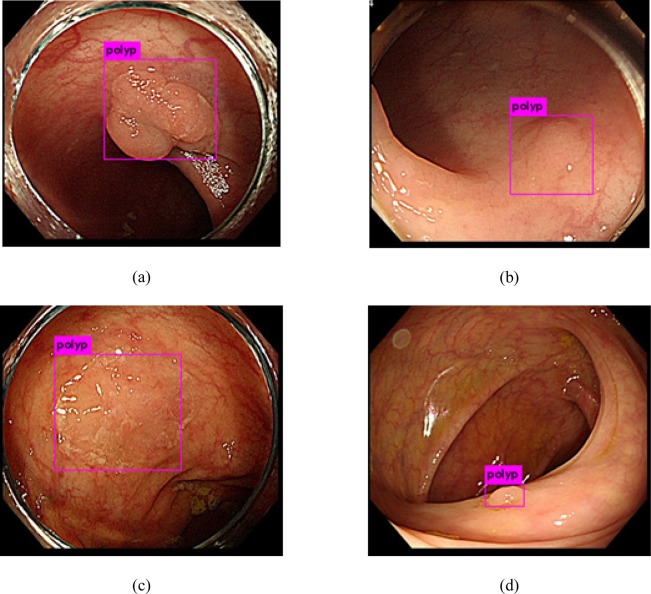
Examples of polyp detection in still-image analysis (dataset A). (**a**) Polypoid polyps, (**b,c**) isochromatic flat polyps, and (**d**) distant, diminutive polyp.

We performed the first validation of the algorithm by analyzing still images from dataset A. The algorithm achieved a per-image sensitivity of 96.7% for the detection of polyps, with 34 FPs (Table 1). The algorithm detected various types of polyps, including large, small isochromatic, and diminutive polyps (Fig. 1). We performed subgroup analyses to investigate the performance of the algorithm according to the polyp size, morphology, and histology (Table 2). The polyp morphology was categorized according to the Paris classification^12^. The polyp size and histology did not affect the performance of the algorithm with regard to detection and localization. However, the algorithm exhibited a higher detection rate for the polypoid type (98.0%) than for the flat type (89.8%) (Table 2).

**Table 1:** Algorithm performance for validation with datasets A and B.

	Number of true positives	Number of false negatives	Number of false positives	Number of true negatives	Sensitivity, %
Dataset A	1305	44	34	NA	96.7
Dataset B	577	63	10	NA	90.2

**Table 2:** Subgroup analysis for true positives and false negatives according to the polyp size, morphology, and histology in validation dataset A.

Polyp characteristics		Total number of frames with polyps	True positive, number (%)	False negative, number (%)
Size	<1 cm	985	961 (97.6)	24 (2.4)
	≥1 cm	364	346 (95.1)	18 (4.9)
Morphology*	I	1152	1129 (98.0)	23 (2.0)
	II	157	141 (89.8)	16 (10.2)
	Laterally spreading tumor	40	37 (92.5)	3 (7.5)
Histology	Tubular adenoma	998	974 (97.6)	24 (2.4)
	Hyperplastic polyp	143	137 (95.8)	6 (4.2)
	Sessile serrated polyp	180	169 (93.9)	11 (6.1)
	Cancer	28	28 (100)	0 (0.0)

*Morphology was classified according to the Paris classification.

We performed external validation of the algorithm using dataset B, to evaluate the generalizability of the algorithm. The algorithm exhibited a per-image sensitivity of 90.2%, with 10 FPs (Table 1).

We performed the third validation of the algorithm using 7 colonoscopy videos with 26 histologically confirmed polyps (dataset C) under real-world colonoscopy-mimicking conditions. Expert endoscopists reviewed all frames of the videos and recorded the ground truth for each frame, i.e., whether each frame included a polyp. The algorithm achieved a per-polyp sensitivity of 100%. The per-image sensitivity was 87.7%, with an accuracy of 87.7% and an FP rate of 12.5%. To reduce the number of FPs, we used a median filter. Table 3 presents the sensitivity and FP rate of the algorithm with respect to the window size. The median filter with a window size of 13 yielded the best performance: an overall per-image sensitivity of 89.9%, a FP rate of 6.3%, and an accuracy of 93.4% (Table 3). We also evaluated the sensitivity of the “first encounter” (88.9%), which represents all the frames from the very first appearance of a polyp. For a median filter with a window size of 13, the algorithm determines the presence of a polyp depending on the median value of probabilities of polyp presence among 13 consecutive frames. The detailed polyp characteristics for dataset C are presented in Supplementary Table S1.

**Table 3:** Sensitivity and false-positive rate of the validation/fine-tuning dataset according to the window size.

	Window size										
	1	3	5	7	9	11	13	15	17	19	21
**Overall polyp tracking**											
Sensitivity (%)	87.7	88.9	89.4	89.6	89.7	89.8	89.9	89.8	89.8	89.8	89.8
AUC	0.877	0.897	0.906	0.91	0.913	0.916	0.918	0.919	0.92	0.921	0.923
**First-encountered polyp detection**											
Sensitivity (%)	87.3	88.1	88.6	88.9	88.8	88.8	88.9	88.5	88	87.6	87.6
AUC	0.875	0.893	0.902	0.907	0.908	0.911	0.913	0.912	0.911	0.911	0.911
False-positive rate (%)	12.3	9.5	8.3	7.6	7.1	6.7	6.3	6	5.8	5.6	5.4

AUC: area under curve

### Final performance test of algorithm using 15 unaltered colonoscopy videos

**Figure 2:**
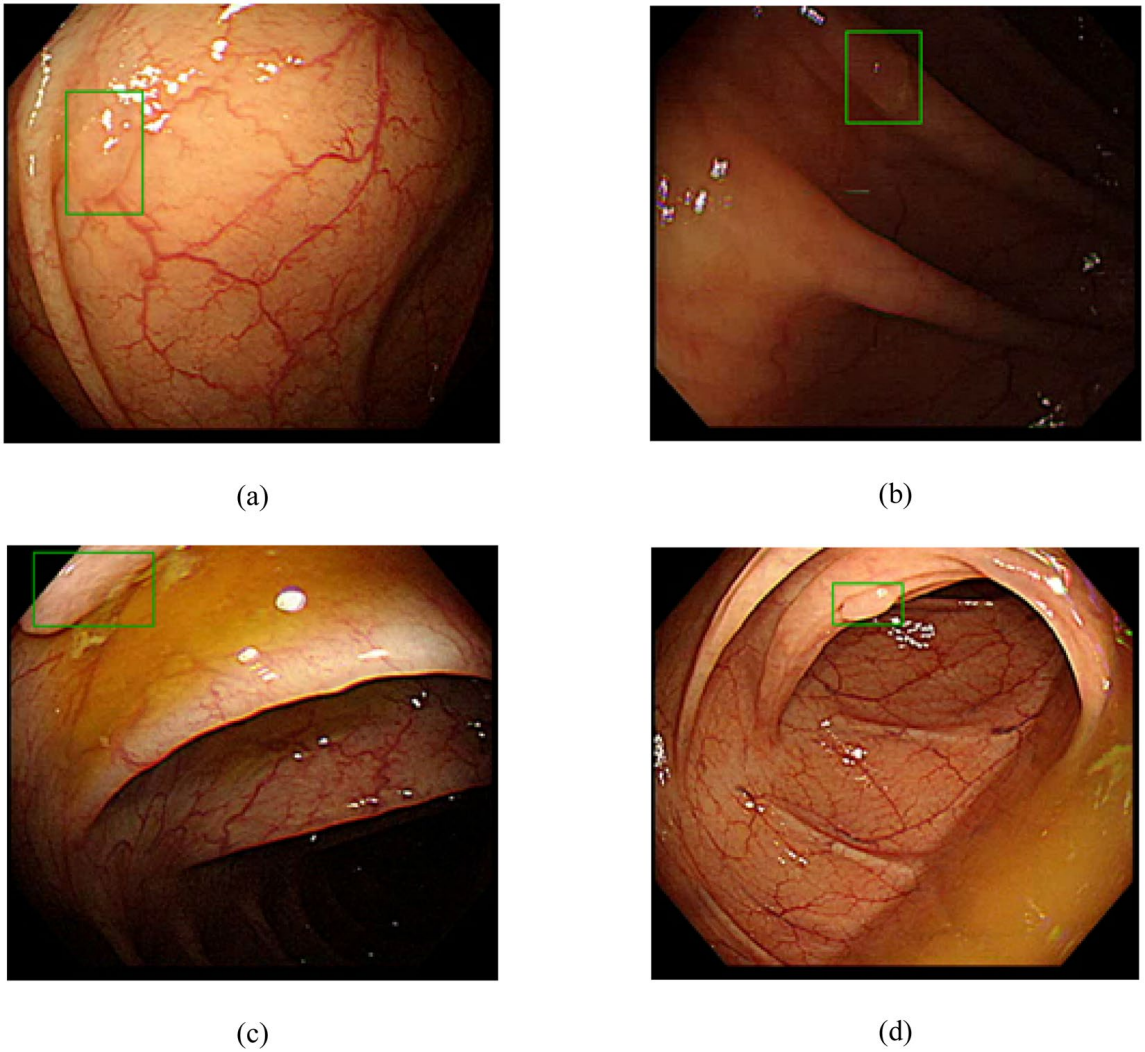
Examples of polyp detection in video-image analysis (dataset D). Green boxes show polyps detected by algorithm. (**a,b**) Polyps detected under various light conditions. (**c**) Partially appearing polyp detected by the algorithm. (**d**) Diminutive polyp detected under suboptimal bowel preparation.

**Figure 3:**
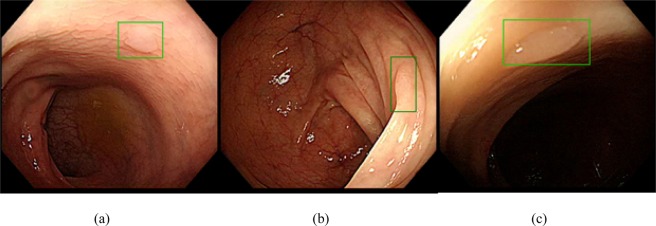
Examples of additional polyps detected by the algorithm (shown in green boxes).

To validate the practical usefulness of the algorithm in real-world colonoscopy, the algorithm was used to analyze 15 unaltered colonoscopy videos (~242,344 frames, 135 min). The algorithm with a median filter having a window size of 13 detected 45 polyps including all 38 polyps originally detected by the endoscopists during the colonoscopy. (Fig. 2, Video S1) Interestingly, the algorithm detected seven additional highly probable colon polyps that were not found by the endoscopists (Fig. 3, Supplementary Table S2). The median size of these polyps was 2 mm (range, 2–3 mm). Two polyps were detected in the ascending colon and five polyps were detected in the sigmoid colon and rectum. Out of these seven polyps, five were polypoid and two were flat. The per-image sensitivity and FP rate of the algorithm were 89.3% and 8.3%, respectively, and the average number of FPs per video was 19. When we increased the window size to 29, the algorithm detected 44 of 45 polyps, with an average of 9 FPs per video (Supplementary Table S2, Table 4). The per-image sensitivity and FP rate were 88.3% and 6.2%, respectively. For the window size of 29, the algorithm detected a polyp when the probability of polyp presence in >15 frames (≥0.5 s) among the 29 frames of the window box exceeded 40%.

**Table 4:** Algorithm performance for two different window sizes in analysis of 15 unaltered colonoscopy videos (dataset D).

Colonoscopy video ID	Total polyps found by endoscopists	Window size = 13			Window size = 29		
		Total polyps found by algorithm	Per-image sensitivity (%)	Total false positives	Total polyps found by algorithm	Per-image sensitivity (%)	Total false positives
8	1	1	92.6	23	1	91.7	14
9	3	4	77.2	16	4	70.6	9
10	3	3	62.6	17	3	62.8	7
11	2	2	97.8	16	2	100	6
12	1	2	93.4	20	1	90.3	9
13	1	1	80.5	9	1	82.6	5
14	1	2	88.7	21	2	86.7	7
15	1	1	77.5	21	1	79.8	9
16	2	2	96.1	22	2	96.6	9
17	4	5	97.4	17	5	96.4	3
18	1	1	88.2	27	1	90.7	15
19	6	9	90.6	26	9	90.0	8
20	8	8	91.2	23	8	91.1	19
21	2	2	95.7	23	2	95.2	11
22	2	2	90.1	13	2	88.8	10

ID: identification^10^.

The processing time for each image frame of the algorithm was 0.0149 ± 0.00016 s. The operating speed of the system was 67.16 frames per second (fps).

## Discussion

Our deep-learning algorithm exhibited highly accurate performance in automatic polyp detection. We validated the algorithm using three different datasets: a split-sample internal image dataset, an external image dataset, and a colonoscopy video dataset. Finally, we evaluated the performance of the algorithm using 15 unaltered colonoscopy videos. Owing to the systematic development and validation processes, our study demonstrates the usefulness of the automatic polyp detection algorithm with high confidence.

Several computer-aided techniques have been previously proposed to assist endoscopists in the detection of colon polyps^13–16^. Recently, deep-learning methods have been reported to improve the performance of computer-aided systems^10,11,17^. Out of eight submissions to the MICCAI 2015 Endoscopic Vision Challenge for polyp detection, the most accurate system using convolutional neural networks exhibited a detection accuracy of 89%, which was tested across 18,092 video frames^17^. In the present study, we developed our algorithm using YOLOv2 with >8,000 colon polyp images. Our algorithm demonstrated an accuracy of 93.4% for the validation process with colonoscopy videos, which is comparable to the results of previous studies^9,11^. Furthermore, during the analysis of the unaltered colonoscopy videos, our algorithm detected not only all the polyps that were found by endoscopists during the original colonoscopy but also additional polyps that were not detected by the endoscopists. These findings suggest that our automatic polyp detection algorithm is practical and accurate. In addition, detection of additional polyps by the algorithm may be meaningful in clinical practice in terms of lowering the risk of interval cancer. The feasibility of the algorithm in real-world clinical practice is also supported by its short processing time. Because our algorithm can process images at a speed of 67 fps for polyp detection, it can be employed for real-world colonoscopy with negligible latency, because colonoscopy video encodings usually have standardized rates of approximately 30 fps.

The recognition of the first appearance of a colon polyp is important for automatic polyp detection systems because the shape of a polyp changes continuously depending on the location, air inflation, angle of the scope, and remnant water and/or stool in real colonoscopy procedures. Thus, we carefully labeled the first appearance of a polyp, such as a polyp edge behind the fold or frame and distantly located polyps, so that the algorithm could be trained under conditions similar to those of endoscopists, who recognize polyps at the very first appearance. We believe that this training strategy improved the detectability and sensitivity of the algorithm.

We used the median filter to reduce the number of FPs. The median filter is a nonlinear spatial filter based on order-statistics theory that is particularly effective for eliminating salt-and-pepper noise^18,19^. Median filtering is useful for removing impulse noise, which is similar to our FP patterns. We applied the median filter with the best window size to our algorithm after we evaluated the optimal threshold by testing window sizes of serial odd numbers. As shown in Table 3, the filter showed the best performance with a window size of 13 during the validation analysis with dataset C. Theoretically, the median filter with a window size of 13 has a risk of missing a polyp only if it appears in <7 frames (<0.23 s), which corresponds to a high sensitivity for polyp detection. Therefore, for dataset D, the algorithm with a median filter having a window size of 13 detected all 45 polyps. However, the algorithm exhibited 19 FPs per video, owing to the high sensitivity. When we increased the window size to 29, the number of FPs decreased; 9 were detected per video, with a minimal decrease in the sensitivity (44 of 45 polyps were detected). The modifiable window size of the median filter may be useful because it can be adjusted according to the endoscopist’s preferences and colonoscopy indications. For example, an expert endoscopist may increase the window size to minimize FPs when performing therapeutic colonoscopy procedures such as polypectomy. This is because the previously performed screening colonoscopy may have already found most polyps. Additionally, an inexperienced endoscopist may reduce the window size to maximize the detection sensitivity of the algorithm when performing screening colonoscopy to avoid missing polyps, which is of paramount importance for a successful CRC screening. We consider the adjustability of the window size of the median filter according to the colonoscopy indications to be the point that discriminates our study from previous studies^10,11^. Another strength of our study compared to previous reports^15,16,20^ is the meticulous validation process based on three independent validation datasets and one unaltered video set as a test dataset. Because our diagnostic sensitivity and specificity on the four separate validation and test datasets composed of both image and video sets was relatively consistent, we consider our study to have demonstrated the usefulness and feasibility of the algorithm in real clinical practice with high confidence.

In our study, the sensitivity and specificity were slightly higher in the image analysis than in the video analysis. Similar results were obtained in previous studies^16,20^. Possible explanations include the following: 1) the image resolution of the videos was lower than that of the still images and 2) the quality of certain image frames from the real-time colonoscopy videos was lower than that of the still images because the videos included blurred image frames owing to the motion of the scope, water suction, folds reflexing light, bleeding after biopsy, and fecal residue. This weakness of the algorithm might be addressed by adding sufficient training data that include blurred image frames.

The sensitivity for detecting isochromatic, flat polyps was slightly lower than that for polypoid polyps in the image analysis. Although the performance was similar for the detection of isochromatic flat and polypoid polyps in the video analysis, further investigations with larger video datasets are necessary, because only a small number of isochromatic, flat polyps were included in the video dataset of our study. Interestingly, our algorithm detected all four sessile serrated polyps in the right colon that were detected by endoscopists (Supplementary Table S2). The sizes of these polyps were in the range 4–9 mm. This finding suggests the algorithm could detect sessile serrated polyps quite accurately, which is important in clinical practice in terms of lowering the risk of interval colorectal cancer because missing sessile serrated polyps has been considered an important cause of interval cancer.

This study had several limitations. First, there could be selection bias in the training dataset because training datasets 1 and 2 were retrospectively selected. However, we believe that quality of our test dataset of 15 unaltered colonoscopy videos did not deviate from the quality of usual real-time colonoscopy videos in daily practices in terms of their consecutive manner of collection. Second, all polyps detected additionally by the algorithm were 2–3 mm in diameter. Small polyps less than 5 mm demonstrated advanced histology only in 0–4.3% of the cases^21,22^. In addition, only 1% of small polyps progressed to advanced adenoma for 7.8 years^23^. Thus, the clinical relevance of polyps detected additionally by the algorithm may not be very high, which limits the usefulness of the algorithm in real clinical practice. However, the system may still help inexperienced colonoscopists with a low adenoma detection rate who may even miss large polyps that can be detected by the algorithm. Third, the algorithm initially showed a FP rate of 8.3% in the unaltered colonoscopy videos. However, we could decrease the FP rate to 6.2% by increasing the window size of median filtering without additional training. The FP rate of 6.2% may be comparable to the FP rates of approximately 5% in previous studies although it is still numerically slightly higher^10,11^. We suggest additional training with a larger amount of training data may further improve the FP rates of the algorithm. The FP cases in our study related to endoscopic features such as collapsed mucosa, debris, light reflexed mucosa and polypectomy site, which were similar to those reported in previous studies^10,11^. Fourth, all the image datasets were obtained using the Olympus endoscope system. Thus, our algorithm cannot be applied directly to other equipment, although we believe that the algorithm may function with other endoscope systems after fine-tuning. Finally, we analyzed recorded videos rather than real-time colonoscopies, limiting the applicability of our algorithm in daily clinical practice. Nonetheless, our algorithm can be applied to real-world colonoscopy procedures because of the short processing time and high performance for unaltered videos, which theoretically represent live colonoscopy. Furthermore, we are confident that the applicability of our algorithm to real-time colonoscopy is supported by our meticulous validation, which involved four independent datasets including one external dataset. This is because evaluation using external validation is more appropriate than internal cross-validation in terms of overfitting during deep learning.

In conclusion, we developed and validated an automatic colon-polyp detection system using deep learning. Our algorithm showed good performance, as evidenced by the high detection sensitivity and rapid processing. The automatic polyp detection algorithm may contribute to successful colonoscopy procedures by reducing the adenoma miss rates and thereby preventing interval CRC, particularly in cases of inexperienced colonoscopists with low adenoma detection rates. Further clinical validation studies with large external video datasets are warranted to evaluate the generalizability of the algorithm in real-world colonoscopy practice.

## Methods

### Training and development of polyp detection algorithm

#### Training dataset

We used 8,075 image frames from 181 colonoscopy video clips of 103 randomly selected patients who underwent a colonoscopy in the endoscopy unit of Asan Medical Center, Seoul, Korea between May 2017 and February 2018. Colonoscopy images with poor bowel preparation were not included in this study because, in our center, colonoscopy was aborted if the bowel preparation was poor. Every video was clipped from when a polyp first appeared in the visual field until it disappeared from the visual field. All the image frames of the video clips were stored at a resolution of 475 × 420 pixels. The location and dimensions of every polyp were labeled using bounding boxes. The videos in the training dataset were acquired using an Olympus EVIS LUCERA CV 290 processor (Olympus Medical Systems Co., Tokyo, Japan). The training dataset of 181 video clips showed an imbalance between several histological types of polyps; i.e., there was a small proportion of flat, isochromatic polyps such as hyperplastic polyps (HPs) and sessile serrated polyps (SSPs). Therefore, we used a second training dataset with 420 additional images from 203 patients, containing 322 HPs and SSPs. For each frame, we applied a data augmentation by doubling the amount of training data, which included the adjustment of the brightness and contrast, blurring, and sharpening. The characteristics of the included polyps are presented in Table 5. This study was conducted in accordance with the declaration of Helsinki. Written informed consents were waived because all the endoscopy images in this study were anonymized before their collection for this study. This study was approved by the institutional review board of the Asan Medical Center (protocol no. 2019–1178).

**Table 5:** Patient demographics and polyp characteristics for the training, validation, and test datasets.

	Training dataset		Validation dataset			Test dataset
	Dataset 1	Dataset 2	Dataset A	Dataset B	Dataset C	Dataset D
Purpose	Initial training of algorithm		Validation of developed algorithm			Final testing of algorithm performance
Data source	Endoscopy unit of AMC	Endoscopy unit of AMC	Endoscopy unit of AMC	CVC-Clinic database	Health screening & promotion center of AMC	Health screening & promotion center of AMC
Data content	8,075 polyp images from 181 colonoscopy videos of 103 patients	420 colonoscopy images with 322 HP or SSP from 203 patients	1,338 colonoscopy images with 1,349 polyps from 879 patients	612 colonoscopy polyp images	7 colonoscopy videos with 26 polyps (~108,778 frames) from 7 patientsPolyp images: 7,022No polyp images: 101,756	Total of 134 min of 15 unaltered colonoscopy videos (242,344 frames) from 15 patients
**Patient demographics**						
Male, number (%)	65 (63.1)	123 (60.5)	565 (64.3)		6 (85.7)	13 (86.7)
Age (years)	59.5 ± 12.1	60.0 ± 12.1	61.6 ± 11.2		47.1 ± 7.6	53.7 ± 8.0
**Polyp characteristics**						
Histology, number (%)	TA, 120 (66.3)	HP, 167 (51.9)	TA, 998 (73.9)		TA, 14 (53.8)	
	HP, 20 (11.0)	SSP, 155 (48.1)	HP, 143 (10.6)		HP, 7 (26.9)	
	SSP, 13 (7.2)		SSP, 180 (13.3)		SSP, 2 (7.7)	
	TSA, 3 (1.7)		CA, 28 (2.1)		IP, 3 (11.5)	
	IP, 11 (6.1)					
	CA, 11 (6.1)					
	Others, 3 (1.7)					
Location, number (%)	Cecum, 19 (10.5)	Cecum, 34 (10.6)	Cecum, 106 (7.8)		Cecum, 1 (3.8)	
	Ascending, 72 (39.8)	Ascending, 132 (40.9)	Ascending, 477 (35.4)		Ascending, 8 (30.7)	
	Transverse, 24 (13.3)	Transverse, 43 (10.4)	Transverse, 241 (17.9)		Transverse, 10 (38.5)	
	Descending, 20 (11.0)	Descending, 23 (7.1)	Descending, 110 (8.1)		Descending, 1 (3.8)	
	Sigmoid, 26 (14.4)	Sigmoid, 64 (19.9)	Sigmoid, 291 (21.6)		Sigmoid, 3 (11.5)	
	Rectum, 20 (11.0)	Rectum, 26 (8.1)	Rectum, 124 (9.2)		Rectum, 3 (11.5)	
Size,number (%)	≤5 mm, 76 (42.0)	≤5 mm, 174 (54.0)	≤5 mm, 630 (46.7)		≤5 mm, 21 (80.8)	
	6–9 mm, 48 (26.5)	6–9 mm, 67 (20.8)	6–9 mm, 355 (26.3)		6–9 mm, 5 (19.2)	
	≥10 mm, 57 (31.5)	≥10 mm, 81 (25.2)	≥10 mm, 364 (27.0)			
Morphology*, number (%)	I, 133 (72.5)	I, 233 (72.3)	I, 1151 (85.3)		I, 22 (84.6)	
	II, 18 (9.9)	II, 85 (26.4)	II, 158 (11.7)		II, 4 (15.4)	
	LST, 30 (16.6)	LST, 4 (1.2)	LST, 40 (3.0)			

AMC: Asan Medical Center; CA: cancer; IP: inflammatory polyp; HP: hyperplastic polyp; LST: laterally spreading tumor; SSP: sessile serrated polyp; TA: tubular adenoma; TSA: traditional serrated adenoma.

*Morphology was classified according to the Paris classification.

#### Model training

We used the second version of YouOnlyLookOnce (YOLOv2)^24,25^ to develop the polyp detection algorithm using deep learning (Supplementary Fig. S1, Supplementary Table S3). This real-time object detection system is capable of one-shot classification of every object in an image without an attention mechanism. We fine-tuned the Darknet19 model pre-trained on the ImageNet dataset using our training images^26^. Supplementary Table S3 shows the network architecture of YOLOv2^25^. The model first resizes images to 416 × 416 pixels and splits them into S × S grids. Then, it creates B bounding boxes that have confidence in prediction for C classes. Each bounding box consists of five values: (x, y) for coordinates, (w, h) for width and height, and confidence for the class probability of the box. The values of S and B are given as 13 and 5, respectively, in YOLOv2, and we set C as 1 because we were only concerned with one class: the polyp. Consequently, the shape of the output vector for each grid cell was (5 + C) × B, which was 30 in our case^25^. YOLOv2 offers a multi-scale training method^25^. During the training, for every 10 batches, the input images were resized to a random value selected from the following list of 10 multiples of 32: 320, 352, …, and 608. The total training time was approximately 12 h on our server, which consisted of two Intel(R) Xeon(R) E5–2650 v2 @ 2.60 GHz 8-core central processing units, six 4-GB random-access memories, and an Nvidia GeForce GTX 1080 graphics processing unit (8 GB) machine.

#### Median filtering to reduce flickering in video overlaid by algorithm

When the algorithm detected a polyp, false positives (FPs) were generated as an impulse style in the time domain and appeared as flickering marks during colonoscopy. We applied the median filter, which is useful for removing impulse noise from a signal^19^, as a post-processing method to reduce flickering marks due to FPs. The key idea of the median filter is to run through the signal entry-by-entry, replacing each entry with the median of the neighboring entries. The pattern of neighbors is called the “window.” To determine the optimal window size of the median filter for the best performance, different window sizes were tested, as shown in Table 3.

### Validation of algorithm

The algorithm was validated using two datasets of still images and one video dataset. These validation datasets were completely independent datasets that were not used for model training. Detailed characteristics of the polyps in the datasets are presented in Table 5.

1. **Dataset A**: We used 1,338 still images of 879 randomly selected patients who underwent a colonoscopy in the endoscopy unit of our institution between May 2017 and February 2018 (Table 5). All 1,349 polyps were diagnosed histologically. Images were acquired using an Olympus EVIS LUCERA CV290 processor (Olympus Medical Systems Co., Tokyo, Japan).
2. **Dataset B**: For external validation, we used a public database: the CVC-Clinic database (https://polyp.grand-challenge.org/CVCClinicDB). This database could be freely used without an independent ethical approval according to the relevant guideline because it is an open database of the part of the endoscopic vision challenge^27^. It consisted of 612 polyp images extracted from 29 colonoscopy videos provided by the Hospital Clinic of Barcelona, Spain. These images were acquired using endoscope equipment, i.e., Olympus Q160AL and Q165L (Olympus Medical Systems Co., Tokyo, Japan).
3. **Dataset C**: For sensitivity and specificity analysis of the videos, we used a series of 7 colonoscopy videos with 26 polyps obtained between November 2018 and January 2019 from a health screening and promotion center. The colonoscopy videos were recorded and evaluated at a resolution of 475 × 420 pixels. Every video included the full withdrawal time from cecal intubation to the anus. The procedure frames starting from forceps insertion to completion of the procedure were edited to concisely evaluate the algorithm performance. Among a total of 108,778 frames, 7,022 frames had polyps, and 101,756 did not.

For the image assessment, expert endoscopists carefully rechecked each algorithm-labeled image and recorded the number of correctly labeled polyps, missed polyps, and false detections in non-polyp areas. The endoscopists also reviewed histologic reports of each polyp. For the video assessment, we validated an algorithm with a 40% (or greater) probability of polyp presence. The algorithm prediction of polyps was indicated by a green box in each frame where a polyp was detected. Three expert endoscopists reviewed each frame of the videos, and when two of the three experts agreed on a highly probable colon polyp in the reviewed videos, we counted it as a true colon polyp detected by the algorithm.

### Final performance test using 15 unaltered colonoscopy videos

To test the real-world performance of the algorithm, we used 15 consecutively collected, unaltered colonoscopy videos with polyps (dataset D). Every video included the full withdrawal time from cecal intubation to the anus, without editing. Three expert endoscopists rechecked each frame of the algorithm-predicted videos and determined the number of correctly labeled polyps, missed polyps, and false detections in non-polyp areas.

### Statistical analysis

A true positive (TP) was defined as the algorithm detecting an actual polyp. A false negative (FN) was defined as the algorithm not detecting polyps in an image with polyps. The sensitivity was defined the number of TPs divided by the total number of polyp appearances (TP + FN). A FP was defined as the algorithm detecting a polyp in an image without polyps or identifying the wrong location. A true negative (TN) was defined as the absence of a detection label in an image without polyps. The specificity was defined as the number of TNs divided by the total number of actual images without polyps. The accuracy was defined as (TP + TN) divided by the total number of frames. The per-image sensitivity was defined as the number of image frames in which a polyp was correctly detected by the algorithm divided by the number of overall image frames with a polyp. The per-polyp sensitivity was defined as the number of polyps correctly detected by algorithm with a per-image sensitivity of ≥50% divided by the total number of actual polyps. The area under the curve (AUC) was calculated using the area under the receiver operating characteristic curve. The receiver operating characteristic curve was obtained by plotting the sensitivity with respect to the 1-specificity for all thresholds in the range of 0–1.

## Supplementary information

Supplementary information.

Supplementary video.

## Data Availability

The datasets generated during and/or analyzed during the current study are available from the corresponding author on reasonable request.
