# Supplementary material for: Real-time detection of colon polyps during colonoscopy using deep learning: systematic validation with four independent datasets
Source: Sci Rep. 2020 May 20;10:8379. doi: 10.1038/s41598-020-65387-1 (PMC7239848; doi:10.1038/s41598-020-65387-1)
Supplement: Supplementary file 1 — Supplementary information. [file 41598_2020_65387_MOESM1_ESM.pdf]

Title: Real-time detection of colon polyps during colonoscopy using deep learning: systematic validation with four independent datasets

Ji Young Lee[1], Jinhoon Jeong[2], Eun Mi Song[3], Chunae Ha[3], Hyo Jeong Lee[1], Ja Eun Koo[1], Dong-Hoon Yang[3], Namkug Kim[4*], and Jeong-Sik Byeon[3*]

[1] Health Screening and Promotion Center, Asan Medical Center, Seoul, Republic of Korea

[2] Department of Biomedical Engineering, Asan Medical Institute of Convergence Science and Technology, Asan Medical Center, University of Ulsan College of Medicine, Seoul, Republic of Korea

[3] Department of Gastroenterology, Asan Medical Center, University of Ulsan College of Medicine, Seoul, Republic of Korea

[4] Department of Convergence Medicine, University of Ulsan College of Medicine, Asan Medical Center, Seoul, Republic of Korea

Ji Young Lee and Jinhoon Jeong contributed equally as the first authors.

Jeong-Sik Byeon and Namkug Kim contributed equally as corresponding authors.

Correspondence to:

Jeong-Sik Byeon, MD, PhD

Department of Gastroenterology, Asan Medical Center,

88, Olympic-ro 43-gil, Songpa-gu, Seoul, Republic of Korea (zip code: 05505)

Tel.: 82-2-3010-3905

Fax: 82-2-476-0824

Email: jsbyeon@amc.seoul.kr

Namkug Kim, PhD

Department of Convergence Medicine, Asan Medical Center, University of Ulsan College of Medicine,

88, Olympic-ro 43-gil, Songpa-gu, Seoul, Republic of Korea (Zip code: 05505)

Tel.: 82-2-3010-6573

Email: namkugkim@gmail.com

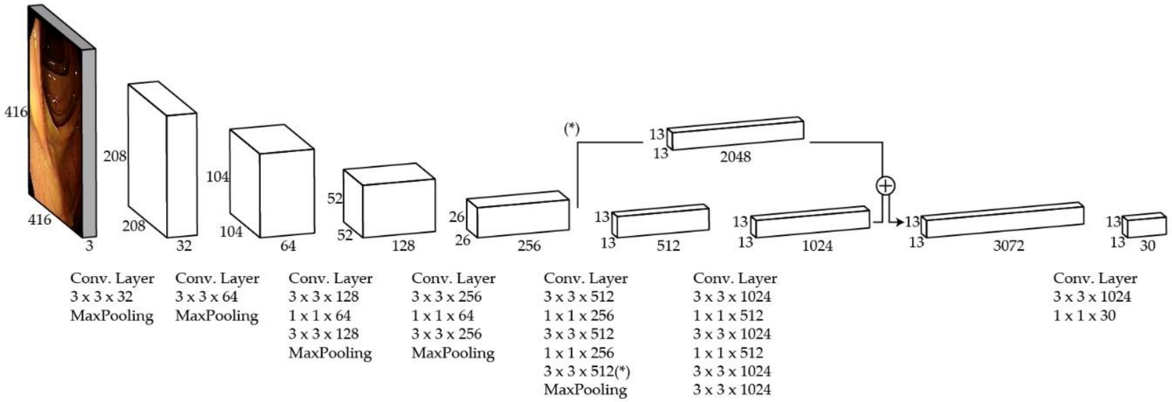

Figure S1. Network structure of YOLOv2.

Table S1. List of all polyps in validation dataset C, including 7 colonoscopy videos

**Colonoscopy ID-polyp ID in corresponding colonoscopy**	**Number of frames with a polyp determined by expert review**	**Number of frames with a polyp determined by algorithm with a median filter (window size 13)**	**Polyp characteristics**			
		Number of frames (%)	Shape	Size (mm)	Location	Histologic diagnosis
No polyp	101,756	96,022 (94.4%)				
1-1	99	86 (86.9%)	II	3	Transverse	HP
2-1	144	141 (97.9%)	I	3	Transverse	TA
2-2	368	300 (81.5%)	I	3	Transverse	TA
3-1	145	145 (100.0%)	I	6	Hepatic flexure	TA
3-2	324	312 (96.3%)	I	4	Transverse	TA
3-3	319	296 (92.8%)	I	4	Transverse	TA
3-4	277	268 (96.8%)	I	3	Transverse	TA
3-5	878	818 (93.2%)	I	3	Sigmoid	HP
4-1	285	214 (75.1%)	I	3	Hepatic flexure	TA
4-2	152	144 (94.7%)	I	4	Transverse	TA
4-3	250	218 (87.2%)	I	5	Transverse	TA
4-4	103	96 (93.2%)	I	3	Rectum	HP
5-1	394	382 (97.4%)	I	4	Cecum	TA
5-2	195	195 (100.0%)	I	4	Transverse	TA
5-3	258	222 (86.0%)	I	3	Sigmoid	TA
5-4	246	197 (80.1%)	II	3	Sigmoid	IP
5-5	56	47 (83.9%)	II	5	Rectum	HP
6-1	382	371 (97.1%)	I	6	Ascending	HP
6-2	814	610 (74.9%)	I	6	Ascending	IP
6-3	119	116 (97.5%)	II	5	Hepatic flexure	IP
6-4	53	48 (90.6%)	I	8	Hepatic flexure	SSP

6-5	236	220 (93.2%)	I	8	Hepatic flexure	HP
7-1	355	339 (95.5%)	I	3	Ascending	TA
7-2	167	148 (88.6%)	I	4	Transverse	SSP
7-3	162	142 (87.7%)	I	4	Descending	TA
7-4	250	237 (94.8%)	I	3	Rectum	HP

HP: hyperplastic polyp; ID: identification; IP: inflammatory polyp; SSP: sessile serrated polyp; TA: tubular adenoma

Table S2. Comparison of polyps found by endoscopists and those detected by algorithm with a median filter for dataset D

Colonoscopy ID-polyp ID	Detection by the algorithm		Shape	Size (mm)	Location	Histologic diagnosis
	Window 13	Window 29				
Polyps found by endoscopists						
8-1	Yes	Yes	I	3	Transverse	TA
9-1	Yes	Yes	I	3	Sigmoid	TA
9-2	Yes	Yes	I	3	Sigmoid	TA
9-3	Yes	Yes	II	3	Sigmoid	HP
10-1	Yes	Yes	II	4	Cecum	HP
10-2	Yes	Yes	I	3	Ascending	TA
10-3	Yes	Yes	I	5	Ascending	SSP
11-1	Yes	Yes	I	6	Cecum	TA
11-2	Yes	Yes	I	3	Sigmoid	TA
12-1	Yes	Yes	I	4	Hepatic flexure	TA
13-1	Yes	Yes	I	4	Ascending	TA
14-1	Yes	Yes	I	4	Transverse	TA
15-1	Yes	Yes	I	3	Ascending	TA
16-1	Yes	Yes	I	5	Ascending	SSP
16-2	Yes	Yes	I	3	Transverse	TA
17-1	Yes	Yes	I	10	Ascending	TA
17-2	Yes	Yes	I	3	Hepatic flexure	IP
17-3	Yes	Yes	I	4	Transverse	TA
17-4	Yes	Yes	I	2	Sigmoid	TA
18-1	Yes	Yes	I	3	Sigmoid	TA
19-1	Yes	Yes	I	4	Transverse	TA
19-2	Yes	Yes	I	5	Splenic flexure	TA

19-3	Yes	Yes	I	2	Descending	IP
19-4	Yes	Yes	I	4	Sigmoid	HP
19-5	Yes	Yes	I	3	Rectum	Lymphoid aggregate
19-6	Yes	Yes	I	3	Rectum	HP
20-1	Yes	Yes	I	7	Cecum	TA
20-2	Yes	Yes	I	12	Cecum	TA
20-3	Yes	Yes	I	4	Hepatic flexure	IP
20-4	Yes	Yes	I	7	Ascending	TA
20-5	Yes	Yes	I	7	Transverse	TA
20-6	Yes	Yes	I	6	Transverse	TA
20-7	Yes	Yes	I	8	Sigmoid	TA
20-8	Yes	Yes	I	12	Sigmoid	TA
21-1	Yes	Yes	I	9	Cecum	SSP
21-2	Yes	Yes	I	5	Ascending	HP
22-1	Yes	Yes	I	4	Ascending	SSP
22-2	Yes	Yes	I	2	Hepatic flexure	HP
Additional polyps detected by algorithm						
Video 9	Yes	Yes	I	2	Ascending	NA
Video 12	Yes	No	I	2	Sigmoid	NA
Video 14	Yes	Yes	I	3	Ascending	NA
Video 17	Yes	Yes	II	3	Sigmoid	NA
Video 19	Yes	Yes	II	3	Sigmoid	NA
	Yes	Yes	I	2	Rectum	NA
	Yes	Yes	I	2	Rectum	NA

HP: hyperplastic polyp; ID: identification; IP: inflammatory polyp; NA: not available; SSP: sessile serrated polyp; TA: tubular adenoma

Table S3. YOLOv2 network architecture

	Layer	Filters	Size/stride	Input	Output
0	Convolutional_1	32	3 x 3 / 1	416 x 416 x 3	416 x 416 x 32
1	Maxpool_1		2 x 2 / 2	416 x 416 x 32	208 x 208 x 32
2	Convolutional_2	64	3 x 3 / 1	208 x 208 x 32	208 x 208 x 64
3	Maxpool_2		2 x 2 / 2	208 x 208 x 64	104 x 104 x 64
4	Convolutional_3	128	3 x 3 / 1	104 x 104 x 64	104 x 104 x 128
5	Convolutional_4	64	1 x 1 / 1	104 x 104 x 128	104 x 104 x 64
6	Convolutional_5	128	3 x 3 / 1	104 x 104 x 64	104 x 104 x 128
7	Maxpool_3		2 x 2 / 2	104 x 104 x 128	52 x 52 x 128
8	Convolutional_6	256	3 x 3 / 1	52 x 52 x 128	52 x 52 x 256
9	Convolutional_7	128	1 x 1 / 1	52 x 52 x 256	52 x 52 x 128
10	Convolutional_8	256	3 x 3 / 1	52 x 52 x 128	52 x 52 x 256
11	Maxpool_4		2 x 2 / 2	52 x 52 x 256	26 x 26 x 256
12	Convolutional_9	512	3 x 3 / 1	26 x 26 x 256	26 x 26 x 512
13	Convolutional_10	256	1 x 1 / 1	26 x 26 x 512	26 x 26 x 256
14	Convolutional_11	512	3 x 3 / 1	26 x 26 x 256	26 x 26 x 512
15	Convolutional_12	256	1 x 1 / 1	26 x 26 x 512	26 x 26 x 256
16	Convolutional_13	512	3 x 3 / 1	26 x 26 x 256	26 x 26 x 512
17	Maxpool_5		2 x 2 / 2	26 x 26 x 512	13 x 13 x 512
18	Convolutional_14	1024	3 x 3 / 1	13 x 13 x 512	13 x 13 x 1024
19	Convolutional_15	512	1 x 1 / 1	13 x 13 x 1024	13 x 13 x 512
20	Convolutional_16	1024	3 x 3 / 1	13 x 13 x 512	13 x 13 x 1024
21	Convolutional_17	512	1 x 1 / 1	13 x 13 x 1024	13 x 13 x 512
22	Convolutional_18	1024	3 x 3 / 1	13 x 13 x 512	13 x 13 x 1024

23	Convolutional_19	1024	3 x 3 / 1	13 x 13 x 1024	13 x 13 x 1024
24	Convolutional_20	1024	3 x 3 / 1	13 x 13 x 1024	13 x 13 x 1024
25	Route			16th	26 x 26 x 512
26	Reorganize		/ 2	26 x 26 x 512	13 x 13 x 2048
27	Route			26th & 24th	13 x 13 x 3072
28	Convolutional_21	1024	3 x 3 / 1	13 x 13 x 3072	13 x 13 x 1024
29	Convolutional_22	30	1 x 1 / 1	13 x 13 x 1024	13 x 13 x 30
